# The New Medical Law

**Published:** 1880-10

**Authors:** 


The Bistoury.

ELMIRA, N. Y., OCTOBER 1880.

The BISTOURY is published Quarterly, upon the first of April, July, October and January, at Fifty Cents a year in advance.

### THE NEW MEDICAL LAW.

The good people of the commonwealth, through their representatives in the legislature, make periodic and laudable attempts at regulating by law, the practice of medicine. Several laws have heretofore been framed and passed without the slightest heed having been paid them either by practitioner or patient, and the present one promises to be equally fortunate.

Such laws, from the very nature of the situation, must become inoperative; it could not well be otherwise. If people, generally accredited intelligent, desire to employ spiritualistic, clairvoyant and magnetic physicians (?)—and the number of such is by no means meagre—if they reap benefit, or *imagine* they do—which is much the same, after all—from such creatures, there should be no law found upon the statute books of a free State to prevent their seeking such aid if they so choose and elect of their own free will.

We may have our own private opinion of the intelligence of a person seeking such aid, and of the downright foolishness and stupidity governing the selection of such a medical advisor, but we have no right to express it to his mortification nor to his hindrance in making the choice.

The present law, regulating the practice of medicine in this state, can never be enforced, because it would prevent a man not only from choosing his medical advisor, but would hinder him in his inalienable right of making an ass of himself if he wanted to,—two liberties secured to him in the constitution of the State.

The law requires that every physician practicing medicine in this State shall go to the county clerk's office in the county where he proposes following his profession, and there register his name, place of nativity and the college of medicine from which he graduated. No persons not a graduate of a legally recognized college of medicine, or licensed from some regular county medical society, is permitted to practice medicine, surgery or obstetrics in this State after the first of the present month, under penalty of fifty dollars for the first offence and one hundred dollars for each subsequent one.

This law is intended to be a protection to the people against quackery and the blunders of ignorant practitioners of medicine. However laudable the intentions of the framers of this law, and however desirous they may have been to protect the good people from blundering prescribers of medicine, the method adopted will never secure to them their cherished desire. Physicians—those *legally* qualified to practice medicine—graduates of accepted medical colleges—are not always truly competent to treat the sick. We can all recall one or more so-called physicians—members of medical societies and generally recognized practitioners of medicine who are also quite as well known for their utter incapability and stupidity as followers of their mischosen profession. To choose between these regular physicians and the irregular pretenders would be hazardous; and the law utterly fails in shielding us from both.

There can be no law framed that will protect the people from incompetent prescribers of medicine. Every man must be his own judge of the capability and desirability of the man he wishes to prescribe for him. To interfere with such choice is contrary to our ideas of independence. As well might you compel us to patronize certain churches and purchase groceries of designated tradesmen. We should be left entirely free to call an educated and skillful physician to our bedside when sick, or a blundering, ignorant clarivoyant, if it gives us pleasure or imagined relief so to do. The profession of medicine can never be elevated, its votaries never made conspicuous, save through genuine merit. The remedy for quackery will be found only in higher medical education. Let the standard for graduation be placed much higher, so that our colleges will become something more than diploma shops.

To graduate in medicine now-a-days, has become much too easy of accomplishment. Too many medical colleges are found competing for pupils, and in their strife require so little of educational attainment upon entering and but little more upon leaving their halls, that it has come to pass the average layman is unable to designate between a graduated physician and an ordinary adventurer in medicine.

Under this condition of affairs the law works no good in trying to suppress quackery, but rather invests it with an importance that it would not otherwise have attained.

